# Association between satellite-detected tropospheric nitrogen dioxide and acute respiratory infections in children under age five in Senegal: spatio-temporal analysis

**DOI:** 10.1186/s12889-022-12577-3

**Published:** 2022-01-26

**Authors:** Ayako Kawano, Yoonhee Kim, Michelle Meas, Karen Sokal-Gutierrez

**Affiliations:** 1 School of Public Health, University of California Berkeley, 2121 Berkeley Way, Berkeley, CA 94704 USA; 2 Department of Global Environmental Health, Graduate School of Medicine, The University of Tokyo, 7-3-1 Hongo, Bunkyo-ku, Tokyo, 113-0033 Japan

**Keywords:** Nitrogen dioxide (NO_2_), Acute respiratory infections, Sentinel-5P, Sub-Saharan Africa

## Abstract

**Background:**

There is growing evidence to suggest that exposure to a high concentration of nitrogen dioxide (NO_2_) can lead to a higher incidence of Acute Respiratory Infections (ARIs) in children; however, such an association remains understudied in Sub-Saharan Africa due to the limited availability of exposure data. This study explored this association by using the satellite-detected tropospheric NO_2_ concentrations measured by Sentinel-5 Precursor and ARI symptoms in children under age five collected in the Demographic and Health Survey (DHS) in Senegal.

**Methods:**

We matched the daily tropospheric NO_2_ exposure with the individual ARI symptoms according to the DHS survey clusters spatially and temporally and conducted a logistic regression analysis to estimate the association of exposure to NO_2_ with ARI symptoms in two preceding weeks.

**Results:**

We observed a positive association between exposure to continuous levels of NO_2_ and ARI symptoms after adjusting for confounders (OR 1.27 per 10 mol/m^2^, 95% CI: 1.06 – 1.52). When the association was further examined by quartile exposure categories, the 4th quartile category was positively associated with symptoms of ARI after adjusting for confounders (OR 1.71, 95% CI: 1.08—2.69). This suggests that exposure to certain high levels of NO_2_ is associated with the increased risk of children having symptoms of ARI in Senegal.

**Conclusions:**

This study highlights the need for increased research on the effects of ambient NO_2_ exposure in Africa as well as the need for more robust, ground-based air monitoring in the region. For a country like Senegal, where more than 90% of the population lives in areas that do not meet the national air quality standards, it is urgently required to implement air pollution prevention efforts to protect children from the health hazards of air pollution.

## Highlights

- Using the satellite-detected tropospheric NO_2_ concentrations, we observed an association between individual exposure to NO_2_ and symptoms of Acute Respiratory Infections (ARIs) among children under 5 years old in Senegal.
- This is the first population-based epidemiological study conducted in Senegal that investigated the adverse health effect of early childhood exposure to ambient concentrations of NO_2_ on ARIs in children.

## Introduction

Nitrogen dioxide (NO_2_) is an air pollutant that is primarily produced from the burning of fossil fuels. Evidence suggests that exposure to high levels of NO_2_ can irritate airways in the respiratory tract [1]. This can exacerbate respiratory diseases, particularly asthma, leading to respiratory symptoms, hospital admissions, and visits to emergency rooms [1]. Notably, growing evidence has suggested that exposure to a high concentration of NO_2_ can lead to higher incidence of Acute Respiratory Infections (ARIs) in children [2]. Children are generally at greater risk for the adverse health effects of NO_2_ due to physiological differences between children’s respiratory systems compared to adults, including taking more frequent breaths, having smaller airways, and being more sensitive to toxins. [3]

ARIs are a leading cause of death among children less than 5 years old [2]. In 2015, the African region experienced the highest burden of ARI-related deaths among children younger than 5 years compared to other regions in the world [4].Similarly, a systematic review study that estimated the number of cases, severe cases, and deaths attributable to childhood pneumonia in 2010 also concluded that the African region experienced the highest burden of morbidity and mortality from childhood pneumonia [5]. In Senegal, a low-income country in West Africa, ARI symptoms under age 5 years was estimated at 16.5%, which was higher than other Sub-Saharan African countries including Cote d’ Ivoire (12.1%) and Cameroon (11.5%) [6]. Senegal has undergone rapid urbanization in recent years and experienced the significant emission of air pollutants including NO_2_, resulting from the uncontrolled industrialization and old fleets of commercial vehicles characterized by inefficient combustion technologies [7]. Consequently, more than 90% of populations live in an area that does not meet the air quality guideline recommended by the World Health Organization (WHO) or the national air quality standard called NS 05–062 [8, 9], in which both standards set the same limit for NO_2_: 40 µg/m^3^ for annual mean and 200 µg/m^3^ for one-hour mean. [9]

Despite the high prevalence of ARIs and plausible correlation between high concentrations of NO_2_ and episodes of ARIs, epidemiological studies for an association between NO_2_ and incidence of ARIs in children remains understudied and consequently underappreciated in Sub-Saharan Africa, including Senegal. One of the reasons is the lack of resources for exposure data collection. Continuous air quality monitoring hardly occurs in Sub-Saharan Africa because most countries do not have a ground monitoring system for ambient air pollution in place [8]. Even when a ground monitoring system exists, a network is usually sparse especially in the rural areas and those monitors are rarely the regulatory reference-grade equipment [8].Given the restriction in reliable exposure data collection, most previous studies that investigated the adverse health effects of ambient air pollution in Sub-Saharan Africa gathered exposure data through independent research teams during brief campaigns or research projects covering small geographic areas. [8]

However, the state-of-the-art applications in recent years enable easier access to digital data in the public health and epidemiology studies such as satellite data, social media data, electronic health records, and mobile phone data [10]. In particular, the use of satellite-detected air pollution data has drawn attention of many researchers as an increasing number of studies have investigated changes in air pollution during the COVID-19 pandemic and its impact on population health [11–14]. For instance, one study examined the changes in NO_2_ emissions during the pandemic in China, Spain, France, Italy, and USA using the multisource satellite data released by National Aeronautics and Space Administration (NASA) and European Space Agency (ESA) [12]. Another study assessed the impact of lockdown on the air quality index and its association with mortality in India using the satellite-based air pollution data. [14].

In this study, we aim to conduct the spatio-temporal analysis of an association between exposure to NO_2_ and ARI symptoms in Senegal by using the satellite-detected exposure data and nationally representative health survey data. To our knowledge, this is the first study to use satellite-detected air pollution data to examine the adverse health effects of exposure to NO_2_ concentrations among children in Senegal.

## Materials and methods

### Study area

Senegal is located in the western part of Africa in latitudes 12° and 17°N, and longitudes 11° and 18°W. The country is divided into 14 regions and has a total area of 196,190 km^2^.

### The Demographic and Health Survey (DHS) data

The DHS Senegal is a national household survey that provides information to monitor population and health situation in Senegal. The sample is based on a stratified two-stage cluster design and drawn to be representative at the national, regional, and residence level [15]. The survey was conducted by trained enumerators using structured questionnaires. Interviewers visited only preselected households and no replacement of the preselected households was allowed.

We obtained the DHS Senegal 2019 dataset conducted from April to December 2019, which includes 4,538 households from 341 clusters across the country. In the DHS survey, census Enumeration Areas (EAs) generally become the survey clusters [16]. In urban areas, an EA can be a city block or apartment building while in rural areas it is typically a village or group of villages [16]. To protect the confidentiality of respondents, a cluster is assigned the geo-coordinates of the center of the sampled EA, a type of aggregation [16]. Secondly, georeferenced coordinates of cluster locations are displaced 0 – 2 km in urban areas and 0 – 5 km in rural areas with 1% displaced 0 – 10 km [16]. The displacement is a random direction and random distance process.

**Fig. 1:**
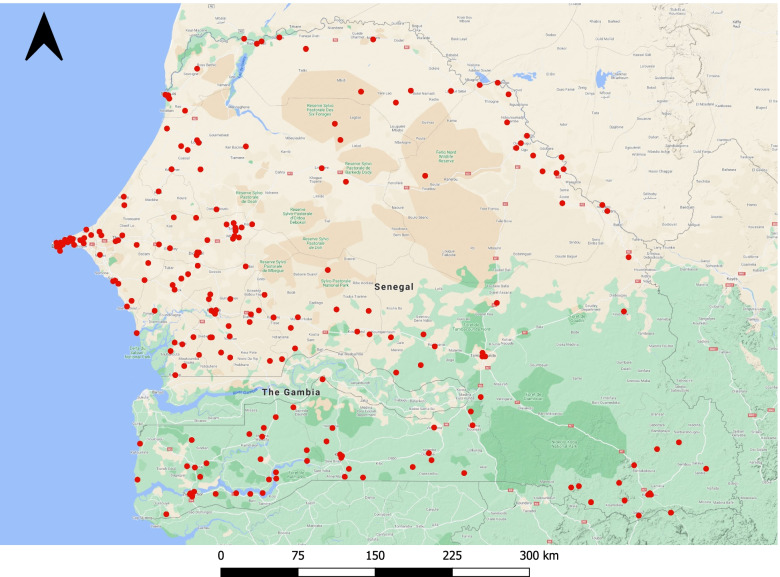
Cluster locations of DHS Senegal 2019 (*n* = 214)

In this study, we extracted the eligible samples from the DHS database including 4,220 children under age 5 living in 214 survey clusters (Fig. 1). The symptoms of ARI were reported by mothers whether their children had short, rapid breathing which was chest-related and/or difficult breathing which was chest-related in the past 2 weeks preceding the survey. The presence of symptoms was assigned the value 1 and otherwise 0. Also, we identified some individual-level potential confounders [2, 3, 17, 18] available in the DHS Senegal 2019 dataset: child’s age (month), area of residence (rural vs. urban), wealth index (rich, middle, or poor), and maternal education level (low vs. high). The wealth index was originally classified into poorer, poorest, middle, richer, and richest, and we re-classified it as poor, middle, and rich. The maternal education level was categorized into low if a mother did not receive any education or attended only preschool or primary school, and high if a mother attended secondary school or higher.

### Spatial distribution of NO_2_

**Fig. 2:**
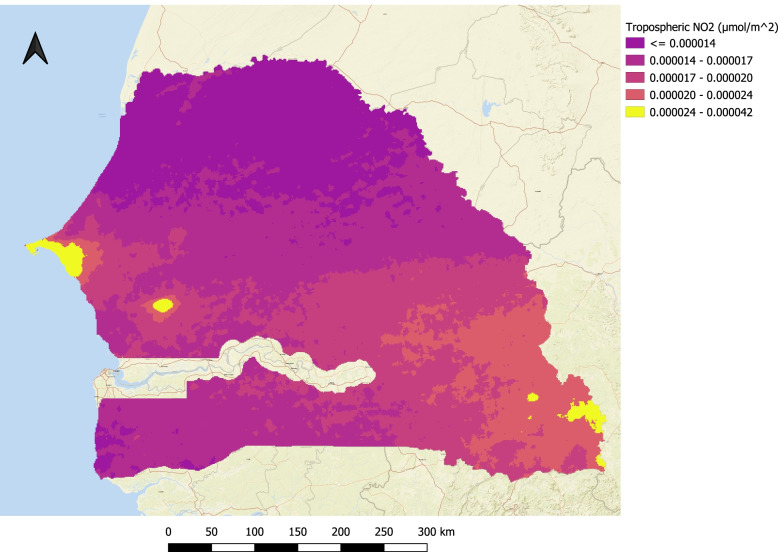
Distribution of mean tropospheric NO_2_ concentrations over Senegal

The tropospheric NO_2_ concentrations were collected from the Sentinel-5 Precursor (Sentinel-5P) space-borne satellite using the Google Earth Engine API. The Sentinel-5P is operated and managed by the European Commission under the “Copernicus” program and has spatial resolution of 7 × 3.5 km^2^. The satellite operates in a sun-synchronous orbit at 824 km and an orbital cycle of 16 days. The satellite carries a TROPOspheric Monitoring Instrument (TROPOMI) which provides a near-global coverage of air pollution caused by NO_2_ and other pollutants such as O_3_, SO_2_, CO, CH_4_, CH_2_O, and aerosols [19]. Fig. 2 shows the spatial distribution of mean tropospheric NO_2_ concentrations over Senegal between April and December 2019.

In this study, we used the tropospheric NO_2_ concentrations collected from the Sentinel-5P as an approximation of ground-level NO_2_ concentrations across the country. Although they are not perfectly matched each other in terms of values, the ground-based validation of Sentinel-5P concluded that the tropospheric NO_2_ had a negative median bias of 23% for low ground-level NO_2_ and 37% for high ground-level NO_2_ [20]. For extremely polluted sites, a negative median bias over 50% was observed [20]. Additionally, another study that conducted a comparison between satellite-based TROPOMI NO_2_ products and ground-based observations revealed high correlation (*r* = 0.68) [21]. Based on the conclusions made by these validation studies, it appeared reasonable to use the satellite-based tropospheric NO_2_ concentrations for this study.

During the exposure data collection, the present study matched the satellite-detected tropospheric NO_2_ concentrations with the cluster locations provided in the DHS dataset spatially and temporally. Data collection and spatial matching was carried out using the Google Earth Engine API. At first, we constructed a buffer zone of 2 km around each reported DHS urban cluster location and 5 km around each reported DHS rural cluster location, and calculated the mean tropospheric NO_2_ level in this buffer zone. Secondly, the exposure data was temporally linked with each respondent of DHS survey using the individual interview date. Since the ARI symptoms were reported by mothers whether a child had symptoms in the past 2 weeks preceding the survey, we calculated the mean tropospheric NO_2_ values of 2 weeks preceding the interview date for each respondent.

### Meteorological data

We identified additional confounders such as mean temperature and relative humidity, and collected them from another satellite data called Global Forecast System (GFS) 384-h predicted atmosphere data. GFS is a weather forecast model produced by the National Centers for Environmental Prediction. The 384-h forecasts, with 3-h forecast interval, are made at 6-h temporal resolution [22]. For temperature, the column values of temperature 2 m above ground were extracted, and for relative humidity, the ones of relative humidity 2 m above ground were collected using the Google Earth Engine API. Similar to NO_2_ concentrations, temperature and relative humidity values were matched with the DHS respondents spatially and temporally.

### Data analysis

Firstly, we examined the sample characteristics stratified by whether children had ARI symptoms. The Chi-square test was used for categorical variables and two-sample t-test was used for continuous variables.

We constructed unadjusted and adjusted binary logistic regression models to determine the crude and adjusted Odds Ratio (OR) for prevalence of ARI symptoms by using the continuous variable of NO_2_ concentrations, which was added as a unit of 10 mol/m^2^. ORs would demonstrate the odds of a child having ARI symptoms per 10 mol/m^2^ increase in NO_2_ concentrations. Additionally, we used a quartile categorical variable of different NO_2_ levels to confirm the association. Confounders included child’s age, area of residence, wealth index, maternal education level, mean temperature, and mean relative humidity. Confounders were chosen based on previous literature identifying potential risk factors for ARIs in children under age 5. We also ensured that there does not exist multicollinearity among independent variables included in the adjusted logistic regression model by computing a Variance Inflation Factor (VIF) score.

In the adjusted logistic regression model, we examined the non-linearity of weather variables such as temperature and relative humidity because previous studies identified that these variables often demonstrate a typical association with health outcomes, characterized by non-linear effects [23]. Therefore, we added temperature and relative humidity as polynomial terms in the regression model with different degrees of freedom, and conducted the likelihood-ratio tests for goodness-of-fit of non-linear regression. As a result, the linear term was selected for temperature and the non-linear term with two degrees of freedom was selected for relative humidity.

For sensitivity analysis, we performed the repeated analysis by using the same models with and without influential points of NO_2_ concentrations defined by the 95th and 99th percentiles. For this, we developed unadjusted and adjusted models that exclude NO_2_ concentrations exceeding the 95th and 99th percentiles.

The statistical significance was established by the p-value as well as 95% Confidence Interval (CI). All statistical analyses were carried out with R. This analysis was reviewed by the University of California, Berkeley Institutional Review Board and was considered as not human subjects research because the study was based on a de-identified and anonymous dataset available for secondary analyses.

## Results

Table 1 describes sample characteristics for 4,220 children under 5 years old with and without ARI symptoms. Overall, 4.4% of children reported ARI symptoms in the past 2 weeks preceding the survey. The younger children were more likely to have ARI symptoms (mean age of 26.79 months), while the one of those without symptoms was 30.53 months (*p* = 0.004). For the wealth index, 31.2% of children with ARI symptoms were rich compared to 23.3% for those without ARI symptoms (*p* = 0.036). In addition, the relative humidity in the DHS clusters was higher for children with ARI symptoms (62.52%) than those for children without ARI symptoms (58.92%), with p-value of 0.047. The mean value for NO_2_ was 17.39 mol/m^2^ with no significant difference between the two groups with and without ARI symptoms and ranged from minimum of 8.34 mol/m^2^ to maximum of 69.29 mol/m^2^.

**Table 1:** Maternal and child characteristics of 4,220 children in the DHS Senegal 2019 dataset

Characteristics	Total (*n* = 4,220)	ARI symptoms: Yes (*n* = 186)	ARI symptoms: No (*n* = 4,034)	*p*-value
Child's age in months, mean (SD)	30.37 (± 17.37)	26.79 (± 16.60)	30.53 (± 17.39)	**0.004**
Mother's education—no. (%)				0.297
High	489 (11.6%)	26 (14.0%)	463 (11.5%)	
Low	3731 (88.4%)	160 (86.0%)	3571 (88.5%)	
Wealth index—no. (%)				**0.036**
Rich	998 (23.6%)	58 (31.2%)	940 (23.3%)	
Middle	795 (18.8%)	28 (15.1%)	767 (19.0%)	
Poor	2427 (57.5%)	100 (53.8%)	2327 (57.7%)	
Residence—no. (%)				0.805
Urban	1259 (29.8%)	57 (30.6%)	1202 (29.8%)	
Rural	2961 (70.2%)	129 (69.4%)	2832 (70.2%)	
Temperature, mean (SD)	26.84 (± 3.09)	26.45 (± 2.82)	26.86 (± 3.10)	0.077
Relative humidity, mean (SD)	59.08 (± 24.15)	62.52 (± 24.53)	58.92 (± 24.13)	**0.047**
NO_2_ (mol/m2), mean (SD)	17.39 (± 7.16)	18.59 (± 8.60)	17.33 (± 7.09)	0.052

Note: Boldface indicates statistical significance (*p* < 0.05).Chi-squared test for categorical variables, and two-sample t-test for continuous variables

The results of the binary logistic regression for ARI symptoms are summarized in Table 2. Both unadjusted and adjusted models showed a significant positive association between exposure to continuous levels of NO_2_ concentration and prevalence of ARI symptoms. The estimated crude OR for having ARI symptoms per 10 mol increase of NO_2_ was 1.22 (95% CI: 1.03 – 1.44) and adjusted OR was 1.27 (95% CI: 1.06 – 1.52). When using the quartile exposure categories, the 2nd and 3rd quartile categories were not significantly associated with symptoms of ARI both in the unadjusted and adjusted models. However, the 4th quartile category was positively associated with symptoms of ARI with statistical significance both in the unadjusted model (OR 1.53, 95%CI: 1.00 – 2.32) and the adjusted model (OR 1.71, 95% CI: 1.08—2.69).

**Table 2:** Unadjusted and adjusted OR and 95% CI of NO_2_ exposure with ARI symptoms

	Unadjusted OR (95% CI)	Adjusted OR (95% CI)
Continuous NO_2_ (10 mol/m^2^)	1.22 (1.03—1.44)^a^	1.27 (1.06—1.52)^a^
Categorical		
Q1 (8.34 – 13.52 mol/m^2^)	1 (Reference)	1 (Reference)
Q2 (13.53 – 16.07 mol/m^2^)	1.23 (0.79—1.91)	1.30 (0.83—2.02)
Q3 (16.08 – 18.89 mol/m^2^)	1.16 (0.75—1.81)	1.21 (0.77—1.91)
Q4 (18.90 – 69.29 mol/m^2^)	1.53 (1.00—2.32)^a^	1.71 (1.08—2.69) ^a^

Adjusted for child's age, maternal education, wealth index, residence, mean temperature, and mean relative humidity.^a^*p* < 0.05

Table 3 summarizes the results of the sensitivity analysis. The 95th percentile of NO_2_ concentrations was 28.85 mol/m^2^ and the 99th percentile was 49.43 mol/m^2^. For the unadjusted models, ORs were no longer statistically significant both when continuous levels and quartile categories of NO_2_ were used. On the other hand, for the adjusted models, the results of the sensitivity analysis demonstrated ORs that were consistent with the original model that incorporated all levels of NO_2_ concentrations. For the model excluding the NO_2_ concentrations above the 95th percentile, the adjusted OR for having ARI symptoms per 10 mol increase of NO_2_ was 1.55 (95% CI: 1.03 – 2.33) and for the model excluding the NO_2_ concentrations above the 99th percentile, the adjusted OR was 1.31 (95% CI: 1.02 – 1.67). When quartile exposure categories were used, the 4th quartile category was positively associated with symptoms of ARI with statistical significance in the adjusted model. OR was 1.79 (95% CI: 1.11 – 2.87) for the model excluding the NO_2_ concentrations above the 95th percentile, and 1.67 (95%CI: 1.06 – 2.64) for the model excluding NO_2_ concentrations above the 99th percentile.

**Table 3:** Unadjusted and adjusted OR and 95% CI of NO_2_ exposure with ARI symptoms by sensitivity analysis

	Unadjusted OR (95% CI)	Adjusted OR (95% CI)
Model excluding the NO_2_ above the 95th percentile		
Continuous NO_2_ (10 mol/m^2^)	1.40 (0.96—2.04)	1.55 (1.03—2.33)^a^
Categorical		
Q1 (8.34 – 13.32 mol/m^2^)	1 (Reference)	1 (Reference)
Q2 (13.33 – 15.69 mol/m^2^)	1.24 (0.79—1.96)	1.32 (0.83—2.10)
Q3 (15.70 – 18.28 mol/m^2^)	1.20 (0.76—1.90)	1.26 (0.78—2.01)
Q4 (18.29 – 28.85 mol/m^2^)	1.52 (0.98—2.36)	1.79 (1.11—2.87)^a^
Model excluding the NO_2_ above the 99th percentile		
Continuous NO_2_ (10 mol/m^2^)	1.23 (0.98—1.55)	1.31 (1.02—1.67)^a^
Categorical		
Q1 (8.34 – 13.39 mol/m^2^)	1 (Reference)	1 (Reference)
Q2 (13.40 – 16.06 mol/m^2^)	1.21 (0.78—1.88)	1.29 (0.83—2.01)
Q3 (16.07 – 18.59 mol/m^2^)	1.14 (0.73—1.77)	1.19 (0.75—1.87)
Q4 (18.60 – 49.43 mol/m^2^)	1.47 (0.96—2.25)	1.67 (1.06—2.64)^a^

Adjusted for child's age, maternal education, wealth index, residence, mean temperature, and mean relative humidity. ^a^*p* < 0.05

## Discussion and conclusion

The present study conducted the spatio-temporal analysis of an association between individual exposure to NO_2_ and prevalence of symptoms of ARI among children under 5 years old in Senegal by utilizing the satellite data in combination with the nationally representative health survey data. Our analysis concluded that there was a positive association between exposure to NO_2_ and ARI symptoms after adjusting for individual-level and ambient potential confounders. This suggests that exposure to certain high levels of NO_2_ is associated with an increased risk of children having symptoms of ARI in Senegal.

This is the first population-based epidemiological study conducted in Senegal that investigated the adverse health effect of early childhood exposure to ambient concentrations of NO_2_ on ARIs in children. Our findings are consistent with previous time-series studies that linked ambient levels of NO_2_ with an increase in ARIs in children [2]. For example, a study conducted in Mexico City revealed that an increment of 30 ppb in the daily mean NO_2_ level was estimated to increase the number of emergency room visits for ARIs during winter by 23% (95% CI: 13.7% to 33.5%) [24]. Also, a study that examined the acute effect of exposure to NO_2_ on daily emergency department visits for upper respiratory infections among children aged 0–4 years in Georgia, USA concluded that there was a positive association between an interquartile range (IQR) increase in NO_2_ and emergency department visits (RR 1.03, 95%CI: 1.02—1.04) [18]. However, no studies assessed the impact of ambient air pollution on ARIs among children in Sub-Saharan Africa because no reliable exposure data is available, according to the systematic review study [8]. We consider that the present study took a novel approach to fulfill the methodological gap by utilizing satellite-detected NO_2_ data as an approximation of ground level exposure.

Our results contribute to the growing body of research highlighting the dangers of ambient air pollution that follow rapid urbanization and industrialization. The WHO estimates that respiratory tract infections caused by air pollution are responsible for over half a million deaths in children less than five years of age worldwide, contributing to more than half of all deaths from acute lower respiratory infections for those living in Low-and Middle-Income Countries (LMICs) [25]. A systematic review of literature found that 24 studies published between 2015 and 2019 linked increased exposure to NO_2_ to increased childhood respiratory morbidity and mortality for children living in Asian LMICs [26]. An increased ARIs due to an increase in ambient NO_2_ and other pollutants is a particularly salient issue in Africa, where ARIs are the leading cause of death in children under 5 years of age. According to a UNICEF report, despite the increase in regulations regarding air quality, there has been a 60% increase in deaths due to outdoor air pollution in Africa from 1990 to 2017 [27]. Though the health outcome of interest in this study was ARI symptoms, future studies could investigate an association of exposure to NO_2_ with pneumonia and reactive airways disease in children as such diseases also contribute to significant morbidity and mortality in Sub-Saharan Africa. [4]

Urbanization is an important vehicle for socio-economic development. Increased urbanization can often foster increased industrialization, access to education, social mobility, and access to healthcare facilities [28]. However, health detriments due to increasing NO_2_ and other air pollutants can offset or, in some cases, outweigh the benefits of urbanization. Sub-Saharan Africa is facing increasing environmental challenges largely due to rapid urbanization and economic transformation. Further exacerbated by lingering poverty, ambient air pollution is expected to continue to rise in a country like Senegal. For the health of children living in Sub-Saharan Africa, it is essential for policy makers to take necessary measures to mitigate ambient air pollution in parallel with promoting economic transformation in the countries. The primary man-made sources for air pollutants in African cities include transportation, industries that utilize fossil fuels, and outdoor burning of waste [29]. Each of these sources can, and should, be addressed with policy interventions. Improving and expanding access to public transportation can reduce the number of personal vehicles on the road, thus reducing the amount of air pollution per capita in urban areas [30]. Improving city infrastructure to add more sidewalks and bike paths can also reduce the use of personal vehicles by encouraging fuel independent modes of transportation [30]. Industry-level pollution can be addressed by imposing fines on sites that exceed an emissions threshold or by providing tax incentives to companies that produce less emissions. Finally, emissions from uncontrolled outdoor waste burning can be reduced by investing in the creation of recycling plants and other waste management infrastructure. Sub-Saharan African countries require unconventional policy interventions that enable both economic development and environmental control for the better health of children living in the countries.

Nevertheless, we acknowledge several limitations of this study. First, the survey data were collected in the retrospective manner, which may lead to the potential recall bias of respondents who were asked whether their children had symptoms of ARI in the past 2 weeks preceding to survey. It also depends on respondents’ awareness of ARI symptoms – parents who lack such awareness may overlook mild or moderate ARI symptoms. Due to this potential recall bias, disease misclassification may have occurred which could have caused either an over-estimate or under-estimate of association. Secondly, data from Sentinel-5P was released on July 10, 2018, which limited the amount of data available for our analysis. Since 2019 was the only full year of Sentinel-5P data at the time of the study, we could not utilize previous years’ DHS publication data in the analysis. Additionally, the exposure of this study, exposure to NO_2_ concentrations may have been subject to measurement error due to the unavailability of ground-based monitoring of air pollution in Senegal for calibration of the satellite data to ground level exposure. In this regard, a previous study that compared relative risk estimates of mortality for fine particulate matter (PM 2.5) modeled from remote sensing with that for PM 2.5 modeled using ground-level data concluded that relative risk estimated from models using remote sensing data could be biased toward the null or underestimated [31]. Finally, although NO_2_ levels were spatially matched with cluster locations provided in the DHS Senegal 2019 dataset, we could not geographically pair them at the household level because such individual GPS data was not available to protect the confidentiality of respondents. Finally, our analysis did not include the investigation of adverse health effects associated with indoor air pollution. However, growing evidence suggests that indoor air pollution is linked to a broad range of cardiorespiratory, maternal, and pediatric conditions and the highest prevalence of exposure to indoor air pollution is observed in LMICs [32]. It is imperative to acknowledge that reducing indoor air pollution is as essential as reducing outdoor air pollution to improve the health of children in Sub-Saharan Africa.

In conclusion, this study provides significant evidence that exposure to NO_2_ is associated with an increased risk of ARIs in children in Senegal, where adverse health effects caused by ambient air pollution have been understudied due to the limited availability of ground-based air monitoring stations. This study highlights the need for increased research on the effects of ambient NO_2_ exposure in Africa as well as the need for more robust, ground-based air monitoring in the region. Expanding the network of real-time, ground-based monitoring of air quality would make it possible to pinpoint the sources of pollution and provide data to make more informed policy implementations. The paucity of data on exposure and outcome assessments led us to propose that more investments need to be made in establishment of reference-grade monitoring systems as well as reliable registration systems and electronic database of morbidity and mortality in Sub-Saharan African countries. Such investments would also enable researchers to investigate the adverse health effects of ambient air pollution through epidemiological studies that utilize more reliable and less biased exposure and outcome data.

## Data Availability

The DHS Senegal 2019 dataset analyzed during the current study is available from the DHS program website (www.dhsprogram.com). The survey and geographic datasets can be found in the following link: https://dhsprogram.com/data/dataset/Senegal_Continuous-DHS_2019.cfm?flag=0. The tropospheric NO_2_ data analyzed during this study can be found in the Google Earth Engine Data Catalog: https://developers.google.com/earth-engine/datasets/catalog/COPERNICUS_S5P_OFFL_L3_NO2#description. The meteorological data used and analyzed in this study can be also found in the Google Earth Engine Data Catalog: https://developers.google.com/earth-engine/datasets/catalog/NOAA_GFS0P25

## Abbreviations

ARI: Acute Respiratory Infection
CI: Confidence Interval
DHS: Demographic and Health Survey
EA: Enumeration Area
ESA: European Space Agency
GFS: Global Forecast System
IQR: Interquartile Range
LMICs: Low-and Middle-Income Countries
NASA: National Aeronautics and Space Administration
NO_2_: Nitrogen Dioxide
OR: Odds Ratio
Sentinel-5P: Sentinel-5 Precursor
TROPOMI: TROPOspheric Monitoring Instrument
VIF: Variance Inflation Factor
WHO: World Health Organaization

## References

[1] United States Environmental Protection Agency. Basic Information about NO_2_. https://www.epa.gov/no2-pollution/basic-information-about-no2. Published July 6, 2016. Accessed 27 Apr 2021.

[2] Romieu I, Samet JM, Smith KR, Bruce N. Outdoor Air Pollution and Acute Respiratory Infections Among Children in Developing Countries. J Occup Environ Med. 2002;44(7):640–9. 10.1097/00043764-200207000-00010. 12134528

[3] World Health Organization. Regional Office for Europe and European Centre for Environment and Health. Effects of air pollution on children’s health and development: a review of the evidence. https://apps.who.int/iris/handle/10665/107652. Published online 2005. Accessed 27 Apr 2021.

[4] McAllister DA, Liu L, Shi T (2018). Global, regional, and national estimates of pneumonia morbidity and mortality in children younger than 5 years between 2000 and 2015: a systematic analysis. Lancet Glob Health.

[5] Rudan I, O’Brien KL, Nair H (2013). Epidemiology and etiology of childhood pneumonia in 2010: estimates of incidence, severe morbidity, mortality, underlying risk factors and causative pathogens for 192 countries. J Glob Health.

[6] Seidu AA, Dickson KS, Ahinkorah BO, Amu H, Darteh EKM, Kumi-Kyereme A (2019). Prevalence and determinants of Acute Lower Respiratory Infections among children under-five years in sub–Saharan Africa: Evidence from demographic and health surveys. SSM - Popul Health.

[7] Ndong Ba A, Verdin A, Cazier F (2019). Individual exposure level following indoor and outdoor air pollution exposure in Dakar (Senegal). Environ Pollut.

[8] Katoto PDMC, Byamungu L, Brand AS (2019). Ambient air pollution and health in Sub-Saharan Africa: Current evidence, perspectives and a call to action. Environ Res.

[9] Sow B, Tchanche B, Fall I, Souaré S, Mbow-Diokhané A (2021). Monitoring of Atmospheric Pollutant Concentrations in the City of Dakar, Senegal. Open J Air Pollut.

[10] Cossin S, Thiébaut R (2020). Public Health and Epidemiology Informatics: Recent Research Trends Moving toward Public Health Data Science. Yearb Med Inform.

[11] Biswal A, Singh T, Singh V, Ravindra K, Mor S (2020). COVID-19 lockdown and its impact on tropospheric NO2 concentrations over India using satellite-based data. Heliyon.

[12] Muhammad S, Long X, Salman M (2020). COVID-19 pandemic and environmental pollution: A blessing in disguise?. Sci Total Environ.

[13] Goldberg DL, Anenberg SC, Griffin D, McLinden CA, Lu Z, Streets DG (2020). Disentangling the Impact of the COVID-19 Lockdowns on Urban NO2 From Natural Variability. Geophys Res Lett.

[14] Naqvi HR, Datta M, Mutreja G, Siddiqui MA, Naqvi DF, Naqvi AR (2021). Improved air quality and associated mortalities in India under COVID-19 lockdown. Environ Pollut.

[15] U.S. Agency for International Development. Demographic and Health Survey Sampling and Household Listing Manual. https://www.dhsprogram.com/pubs/pdf/DHSM4/DHS6_Sampling_Manual_Sept2012_DHSM4.pdf. Published 2012. Accessed 29 Apr 2021.

[16] U.S. Agency for International Development. Geographic displacement procedure and georeferenced data release policy for the Demographic and Health Surveys. DHS Spatial Analysis Report No. 7. https://dhsprogram.com/pubs/pdf/SAR7/SAR7.pdf. Published 2013. Accessed 30 Apr 2021.

[17] Huangfu P, Atkinson R (2020). Long-term exposure to NO2 and O3 and all-cause and respiratory mortality: A systematic review and meta-analysis. Environ Int.

[18] Darrow LA, Klein M, Flanders WD, Mulholland JA, Tolbert PE, Strickland MJ (2014). Air Pollution and Acute Respiratory Infections Among Children 0–4 Years of Age: An 18-Year Time-Series Study. Am J Epidemiol.

[19] Veefkind JP, Aben I, McMullan K (2012). TROPOMI on the ESA Sentinel-5 Precursor: A GMES mission for global observations of the atmospheric composition for climate, air quality and ozone layer applications. Remote Sens Environ.

[20] Verhoelst T, Compernolle S, Pinardi G (2021). Ground-based validation of the Copernicus Sentinel-5P TROPOMI NO_2_ measurements with the NDACC ZSL-DOAS, MAX-DOAS and Pandonia global networks. Atmospheric Meas Tech.

[21] Ialongo I, Virta H, Eskes H, Hovila J, Douros J (2020). Comparison of TROPOMI/Sentinel-5 Precursor NO_2_ observations with ground-based measurements in Helsinki. Atmospheric Meas Tech.

[22] National Centers for Environmental Information. Global Forecast System. https://www.ncdc.noaa.gov/data-access/model-data/model-datasets/global-forcast-system-gfs. Accessed 3 May 2021.

[23] Gasparrini A, Armstrong B (2010). Time series analysis on the health effects of temperature: Advancements and limitations. Environ Res.

[24] Télles-Rojo MM, Romieu I, Polo-Peña M, Ruiz-Velasco S, Meneses-González F, Hernández-Avila M (1997). Efecto de la contaminación ambiental sobre las consultas por infecciones respiratorias en niños de la Ciudad de México. Salud Pública México.

[25] World Health Organization. Air pollution and child health: prescribing clean air: summary. https://apps.who.int/iris/handle/10665/275545. Published online 2018. Accessed 23 June 2021.

[26] Nhung NTT, Amini H, Schindler C (2017). Short-term association between ambient air pollution and pneumonia in children: A systematic review and meta-analysis of time-series and case-crossover studies. Environ Pollut.

[27] United Nations Children’s Fund. Silent Suffocation in Africa: Air Pollution is a Growing Menace, Affecting the Poorest Children the Most.https://www.unicef.org/media/55081/file/Silent%20suffocation%20in%20africa%20air%20pollution%202019%20.pdf. Published online 2019. Accessed 23 June 2021.

[28] Kelly FJ, Fussell JC (2020). Global nature of airborne particle toxicity and health effects: a focus on megacities, wildfires, dust storms and residential biomass burning. Toxicol Res.

[29] Mbow-Diokhane A. Air Quality in African Cities. In: Mboup G, Oyelaran-Oyeyinka B, eds. Smart Economy in Smart African Cities: Sustainable, Inclusive, Resilient and Prosperous. Advances in 21st Century Human Settlements. Springer; 2019. p. 297–311. 10.1007/978-981-13-3471-9_9

[30] Abera A, Friberg J, Isaxon C, et al. Air Quality in Africa: Public Health Implications. Annu Rev Public Health. 42:193-210. 10.1146/annurev-publhealth-100119-113802 33348996

[31] Jerrett M, Turner MC, Beckerman BS (2017). Comparing the Health Effects of Ambient Particulate Matter Estimated Using Ground-Based versus Remote Sensing Exposure Estimates. Environ Health Perspect.

[32] Lee KK, Bing R, Kiang J (2020). Adverse health effects associated with household air pollution: a systematic review, meta-analysis, and burden estimation study. Lancet Glob Health.

